# Final results on effectiveness and safety of Ibrutinib in patients with chronic lymphocytic leukemia from the non-interventional FIRE study

**DOI:** 10.1007/s00277-024-05666-3

**Published:** 2024-03-06

**Authors:** Caroline Dartigeas, Anne Quinquenel, Loïc Ysebaert, Marie-Sarah Dilhuydy, Bruno Anglaret, Borhane Slama, Katell Le Du, Stéphanie Tardy, Emmanuelle Tchernonog, Hubert Orfeuvre, Laurent Voillat, Stéphanie Guidez, Jean-Valère Malfuson, Sandrine Dupuis, Marine Deslandes, Pierre Feugier, Véronique Leblond, on behalf of the FIRE Investigators Group, Didier Adiko, Philippe Agape, Sophie Auger Quittet, Benoît Bareau, Omar Benbrahim, Philippe Bernard, Charles Bescond, Fontanet Bijou, Laurys Boudin, Sylvie Cailleres, Claire Calmettes, Guillaume Cartron, Régis Costello, Selva David, Jacques Delaunay, Caroline Delette, Sophie Dennetiere, Bernard Drenou, Abderrazak El Yamani, Alain Delmer, Olivier Fitoussi, Emmanuel Fleck, Joël Fleury, Jean Gutnecht, Maya Hacini, Éric Jourdan, Régis Kaphan, Jean-Michel Karsenti, Jean-Luc Labourey, Vincent Launay, Ronan Le Calloch, Isabelle Leduc, François Lefrere, Stevan Le Gall, Marielle Le Goff, Éric Legouffe, Steven Le Gouill, Stéphane Lepretre, Jixing Liu, Carine Luttiau Motard, Marius Moldovan, Lysiane Molina, Isabelle Moullet, Frédéric Peyrade, Philippe Quittet, Daniel Re, Virginie Roland, Damien Roos-Weil, Alain Saad, Hussam Saad, Delphine Senecal, Alexia Thannberger, Catherine Thieblemont, Olivier Tournilhac, Sorin Visanica

**Affiliations:** 1 Hématologie et Thérapie Cellulaire, CHRU Hôpitaux de Tours, 2 Boulevard Tonnellé, 37044 Tours Cedex 9, France; 2 CHU de Reims, Reims, France; 3 IUCT Oncopôle, CHU de Toulouse, Toulouse, France; 4 Hôpital Haut-Lévêque, Bordeaux, France; 5 CH de Valence, Valence, France; 6 CH Henri Duffaut, Avignon, France; 7 Hôpital Privé du Confluent, Nantes, France; 8 CH Annecy Genevois, Annecy, France; 9 CHU de Montpellier, Montpellier, France; 10 CH de Bourg-en-Bresse, Bourg-en-Bresse, France; 11 CH William Morey, Chalon-sur-Saône, France; 12 CHU de Poitiers, Poitiers, France; 13 HIA Percy, Clamart, France; 14 Janssen France, Issy-les-Moulineaux, France; 15 Hôpitaux de Brabois, CHU de Nancy, Nancy, France; 16 AP-HP Hôpital de La Pitié-Salpêtrière, Paris La Sorbonne, Paris, France

**Keywords:** Chronic lymphocytic leukemia, Ibrutinib, Real-world evidence, Effectiveness, Safety

## Abstract

We conducted an observational study (FIRE) to understand the effectiveness and safety outcomes of ibrutinib in patients with chronic lymphocytic leukemia (CLL) in France, after a maximum follow-up of five years. Patients were included according to the French marketing authorization in 2016 (i.e. patients with relapsed or refractory CLL or to previously untreated CLL patients with deletion 17p and/or tumor protein p53 mutations unsuitable for chemoimmunotherapy) and could have initiated ibrutinib more than 30 days prior their enrolment in the study (i.e. retrospective patients) or between 30 days before and 14 days after their enrolment (i.e. prospective patients). The results showed that in the effectiveness population (*N* = 388), the median progression-free survival (PFS) was 53.1 (95% CI: 44.5–60.5) months for retrospective patients and 52.9 (95% CI: 40.3–60.6) months for prospective patients and no difference was shown between the PFS of patients who had at least one dose reduction versus the PFS of patients without dose reduction (*p* = 0.7971 for retrospective and *p* = 0.3163 for prospective patients). For both retrospective and prospective patients, the median overall survival was not reached. The most frequent treatment-emergent adverse event of interest was infections (57.6% retrospective; 71.4% prospective). A total of 14.6% of the retrospective patients and 22.4% of the prospective patients had an adverse event leading to death. Our findings on effectiveness were consistent with other studies and the fact that patients with dose reductions had similar PFS than patients without dose reduction is reassuring. No additional safety concerns than those already mentioned in previous studies could be noticed.

**Trial registration **ClinicalTrials.gov, NCT03425591. Registered 1 February 2018 – Retrospectively registered.

**Supplementary Information:**

The online version contains supplementary material available at 10.1007/s00277-024-05666-3.

## Introduction

Chronic lymphocytic leukemia (CLL) is the most common leukemia in adults in Western countries [1]. In 2019, the global age-standardized incidence rate was 1.28 cases per 100,000 persons [2]. The median age at diagnosis is 70 years old [3] and the disease is more common in male patients (global sex ratio: 1.4 men/women) [2].

A decade ago, targeted therapies have been developed with ibrutinib, a first-in-class, oral, once daily Bruton’s Tyrosine Kinase inhibitor (BTKi). Such therapies started to progressively replace first chemoimmunotherapy for relapsed CLL patients and then in first line treatment. Ibrutinib has been authorized in Europe in October 2014 and commercialized in France since November 21^st^, 2014. Currently, it is indicated in Europe for the treatment of all CLL and Waldenström’s macroglobulinaemia adult patients, and for the treatment of relapsed or refractory (R/R) MCL in adult patients [4, 5].

The efficacy of ibrutinib compared to chemoimmunotherapy-based treatment has been largely demonstrated in several clinical trials. Phase-3 studies (RESONATE-2 and RESONATE) showed that previously untreated patients with CLL and R/R CLL had better progression-free survival (PFS) and overall survival (OS) when treated with ibrutinib than with chlorambucil [6] or ofatumumab [7, 8]. Other trials showed similar results in CLL (ALLIANCE: ibrutinib alone or in combination with rituximab versus bendamustine with rituximab; ILLUMINATE: ibrutinib in combination with obinutuzumab versus chlorambucil with obinutuzumab; HELIOS: ibrutinib in combination with bendamustine and rituximab versus bendamustine and rituximab; and GLOW: first-line fixed-duration ibrutinib in combination with venetoclax versus chlorambucil with obinutuzumab) [9–12].

To complement these clinical trials results, the FIRE study was set up to investigate, in France, in real-life conditions, the effectiveness and safety of ibrutinib treatment in patients with CLL (including small lymphocytic lymphoma (SLL)), along with those with high-risk features (e.g. deletion (del)17p or tumor protein p53 (TP53) mutation; unmutated immunoglobulin heavy chain (IGHV) genes). Results of the second and third interim analyses were previously reported [13, 14]. In the second interim analysis, with a median follow-up of 17.4 months, the findings confirmed effectiveness in R/R patients with high-risk features and did not highlight additional adverse events (AE) than those documented in clinical trials [13, 15]. In the third interim analysis, with a median follow-up of 47.2 months, the results showed that ibrutinib was still an effective treatment for CLL patients and that patients who have received ibrutinib in earlier line of treatment had a better PFS [14]. Again, the effectiveness and safety profiles in this third interim analysis were consistent with the results of clinical trials. In this article, the objective was to report the final results of the FIRE study on effectiveness and safety outcomes for CLL patients, after a maximum follow-up of five years.

## Methods

### Study design

FIRE was a retro-prospective, non-interventional, multicenter study, implemented in France through specialized onco-haematology centres. A total of 65 centres participated in the study. The first CLL patient was included on May 12^th^, 2016, and the last visit of the last CLL patient occurred on July 26^th^, 2022. Patients were recruited in the study for about one year and were followed for up to five years.

Patients could have initiated ibrutinib more than 30 days prior their enrolment in the study and been enrolled regardless of whether or not they were still receiving ibrutinib at the time of inclusion (i.e. retrospective patients), or they could have started ibrutinib between 30 days before and 14 days after their inclusion (i.e. prospective patients). The overall design of the study has been provided in Online Resource 1.

### Study participants

Adults with a confirmed diagnosis of CLL and who initiated ibrutinib therapy on or after November 21^st^, 2014, or who planned to initiate ibrutinib within the next 14 days could participate in the study. Patients were included according to the French marketing authorization in 2016, corresponding either to patients with R/R CLL or to previously untreated CLL patients with del17p and/or TP53 mutations unsuitable for chemoimmunotherapy. Patients who were part of the ibrutinib Temporary Authorization for Use, who participated at the same time in another research study and who did not sign the Informed Consent Form were not eligible.

### Outcomes

The primary outcome was the progression-free survival (PFS). Secondary outcomes included overall survival (OS), treatment responses, duration of response (DOR), time to best response / first response / next treatment, treatment discontinuation (permanent), dose reductions (i.e. temporary reduction followed by a dose increase or another dose reduction, and permanent dose reductions, but no temporary ibrutinib discontinuations followed by a restart at a lower dose), and safety. The definition of the different endpoints is provided in Online Resource 2. The safety analyses included AEs (i.e. untoward medical occurrence after exposure to a medicine, which is not necessarily caused by that medicine [16]), treatment-emergent adverse events (TEAE), treatment-emergent bleeding events and AEs leading to death.

### Data collection

All data were collected through the medical records of the patients. The data were collected at different time points between inclusion and the end of the study (Online Resource 1). For patients who initiated ibrutinib therapy at least 31 days before their enrolment, data were also collected retrospectively except for AEs not related to ibrutinib. All investigators were trained to fill in the Electronic Case Report Form and on the use of the Electronic Data Capture System.

### Sample size

We used the following hypothesis to calculate our sample size: a 30-month PFS rate of 76% [15]. Therefore, the PFS at 24 months was estimated to be 80%. Considering this 24-month PFS rate, a rate of censored patients during the first 24 months of 10% and a Confidence Interval (CI) half-width of 4.1%, 400 CLL patients needed to be included to estimate a two-sided 95% CIs for a PFS rate.

### Data analysis and statistics

The statistical analysis on effectiveness parameters (e.g. PFS, OS, DOR, etc.) was performed on all included patients who met the inclusion and non-inclusion criteria and who took at least one dose of ibrutinib (effectiveness population). The statistical analysis on safety parameters was performed on all included patients who took at least one dose of ibrutinib (safety population).

Demographic information (i.e. age, gender), medical history and comorbidities, treatment history and subsequent treatment were obtained and summarized as frequency and percentage.

All time-to-event variables (i.e. PFS, OS, DOR, time to first response / best response / next therapy) were analysed using standard survival analysis methods, including Kaplan–Meier product-limit survival curve. Responses were assessed by physicians. The median time to event with two-sided 95% CIs was estimated. In addition, the PFS was also analysed by mutation status (i.e. mutated (del17p and/or TP53) vs. not mutated) and by dose reduction (i.e. patients with at least one dose reduction vs. no dose reduction). For the PFS by dose reduction, an exploratory logrank test with a level of significance of p = 0.05 was used to determine the effectiveness of ibrutinib among those who had at least one dose reduction vs. those who did not. All data were analysed by inclusion type (i.e. retrospective / prospective) with SAS® version 9.4 (SAS Institute, North Carolina, USA).

## Results

### Patients’ characteristics at ibrutinib initiation

A total of 388 patients was included in the effectiveness analysis (194 retrospective and 194 prospective) (Table 1). Most patients were male (66.5%), ≤ 75 years old (64.9%) and with an ECOG performance status of 0 or 1 (89.6%). Almost half of the patients (48.5%) had at least one medical history and comorbidity. Of those who underwent molecular and cytogenetic assessment, 58.2% (*N* = 156/268) had del17p and/or TP53 mutation and 30.0% (*N* = 81/270) del11q mutation. The median time between the initial diagnosis and the start of ibrutinib was 7.0 (range: 0.0–35.0) years. Most patients (85.3%, *N* = 331) were R/R patients. Among those who were previously treated, the median number of prior therapies was 2 (range: 1–7). All those who were previously untreated for CLL had del17p and/or TP53 mutations.

**Table 1:** Patient and illness characteristics by type of inclusion (Effectiveness population, *N* = 388)

		RETRO (*N* = 194)	PRO (*N* = 194)	TOTAL (*N* = 388)
**Type of hematologic malignancy, N (%)**	CLL	185 (95.4)	186 (95.9)	371 (95.6)
	SLL	9 (4.6)	8 (4.1)	17 (4.4)
**Demographic data**				
Age at ibrutinib initiation, N (%)	≤ 75 years old	128 (66.0)	124 (63.9)	252 (64.9)
	> 75 years old	66 (34.0)	70 (36.1)	136 (35.1)
Gender, N (%)	Male	122 (62.9)	136 (70.1)	258 (66.5)
	Female	72 (37.1)	58 (29.9)	130 (33.5)
**Clinical assessment at ibrutinib initiation**				
ECOG PS, N (%)^a^	0	79 (53.0)	76 (48.4)	155 (50.7)
	1	56 (37.6)	63 (40.1)	119 (38.9)
	2	11 (7.4)	15 (9.6)	26 (8.5)
	3	3 (2.0)	3 (1.9)	6 (2.0)
**Medical history and comorbidity at ibrutinib initiation**				
At least one medical history or comorbidity, N (%)		95 (49.0)	93 (47.9)	188 (48.5)
Prior bleeding event, N (%)^b^		3 (1.6)	7 (3.7)	10 (2.6)
History of significant cardiovascular disease, N (%)^c^		15 (7.7)	22 (11.5)	37 (9.6)
Ongoing malignancy (other than CLL), N (%)^d^		5 (2.6)	4 (2.1)	9 (2.3)
Ongoing active infection with hepatitis B or C, N (%)^e^		1 (0.5)	1 (0.5)	2 (0.5)
Ongoing autoimmune haemolytic anaemia, N (%)^f^		3 (1.6)	8 (4.2)	11 (2.9)
Ongoing atrial fibrillation, N (%)^c^		4 (2.1)	7 (3.6)	11 (2.8)
Other ongoing cardiovascular disease, N (%)^c^		6 (3.1)	10 (5.2)	16 (4.1)
Ongoing respiratory disease, N (%)^c^		14 (7.2)	16 (8.3)	30 (7.8)
Ongoing uncontrolled active systemic infection or grade 3–4 infection, N (%)^g^		–	2 (1.0)	2 (0.5)
Creatinine clearance < 30 mL/min, N (%)^h^		1 (0.5)	3 (1.6)	4 (1.1)
Creatinine clearance ≥ 30 mL/min and < 70 mL/min, N (%)^h^		39 (21.1)	43 (23.1)	82 (22.1)
**Molecular and cytogenetic at ibrutinib initiation**				
Del17p present and/or mutated TP53, N (%)^i^		83 (59.3)	73 (57.0)	156 (58.2)
Del17p present, N (%)^j^		70 (45.2)	52 (36.4)	122 (40.9)
Del13q present, N (%)^k^		51 (41.1)	40 (37.7)	91 (39.6)
Del11q present, N (%)^l^		44 (30.8)	37 (29.1)	81 (30.0)
Trisomy 12 present, N (%)^m^		27 (22.1)	25 (27.2)	52 (24.3)
TP53 mutated, N (%)^n^		59 (43.7)	50 (42.4)	109 (43.1)
IGHV unmutated, N %)^o^		39 (81.3)	21 (72.4)	60 (77.9)
Complex karyotype, N (%)^p^		60 (51.7)	62 (62.6)	122 (56.7)
**Treatment history at ibrutinib initiation**				
Median time between initial diagnosis and ibrutinib initiation, median (range), years		6.48 (0.0–35.0)	7.24 (0.1–27.6)	6.98 (0.0–35.0)
Median number of prior therapy among those previously treated (range)		2 (1–7)	2 (1–6)	2 (1–7)
Number of prior line of therapies, N (%)	0	24 (12.4)	33 (17.0)	57 (14.7)
	1	72 (37.1)	68 (35.1)	140 (36.1)
	2	56 (28.9)	55 (28.4)	111 (28.6)
	≥ 3	42 (21.6)	38 (19.6)	80 (20.6)
Type of therapy previously received, N (%)	Combination therapies	113 (66.5)	118 (73.3)	231 (69.8)
	Monotherapies	13 (7.6)	5 (3.1)	18 (5.4)
	Both	44 (25.9)	38 (23.6)	82 (24.8)
Patients with prior stem cell transplant, N (%)^q^		4 (2.2)	8 (4.9)	12 (3.4)
Treatment-free period between last therapy and ibrutinib initiation, N (%)^r^	< 36 months	118 (76.1)	97 (66.9)	215 (71.7)
	≥ 36 months	37 (23.9)	48 (33.1)	85 (28.3)
**Concomitant medications**				
At least one concomitant systemic anti-cancer therapy		9 (4.6)	17 (8.8)	26 (6.7)
At least one antithrombotic therapy		40 (20.6)	46 (23.7)	86 (22.2)
**Subsequent treatment**		*N* = 198^ s^	*N* = 196^ s^	*N* = 394^ s^
Initiation of a subsequent treatment, N (%)		83 (41.9)	65 (33.2)	148 (37.6)
	Chemotherapy / Immunochemotherapy	21 (25.3)	18 (27.7)	39 (26.4)
	Venetoclax ± Rituximab	45 (54.2)	32 (49.2)	77 (52.0)
	Ibrutinib^t^	12 (14.5)	12 (18.4)	24 (16.2)
	Idealisib – R	3 (3.6)	3 (4.6)	6 (4.1)
	Allotransplantation	2 (2.4)	-	2 (1.4)

The percentages were presented on non-missing values. They are rounded and sometimes do not add to 100%

*Abbreviations*: *CLL* Chronic Lymphocytic Leukaemia, *Del* Deletion, *ECOG PS* Eastern Cooperative Oncology Group Performance Status, *IGHV* Immunoglobulin Heavy Chain Variable Region, *PRO* Prospective, *RETRO* Retrospective, *SLL* Small Lymphocytic Lymphoma, *TP53* Tumour Protein P53

^a^ Missing data: 45 retrospective, 37 prospective, 82 total

^b^ Missing data: 1 retrospective, 5 prospective, 6 total

^c^ Missing data: 2 prospective, 2 total

^d^ Missing data: 1 retrospective, 3 prospective, 4 total

^e^ Missing data: 9 retrospective, 7 prospective, 16 total

^f^ Missing data: 2 retrospective, 5 prospective, 7 total

^g^ Missing data: 2 retrospective, 3 prospective, 5 total

^h^ Missing data: 9 retrospective, 8 prospective, 17 total

^i^ Missing data: 54 retrospective, 66 prospective, 120 total

^j^ Missing data: 39 retrospective, 51 prospective, 90 total

^k^ Missing data: 70 retrospective, 88 prospective, 158 total

^l^ Missing data: 51 retrospective, 67 prospective, 118 total

^m^ Missing data: 72 retrospective, 102 prospective, 174 total

^n^ Missing data: 59 retrospective, 76 prospective, 135 total

^o^ Missing data: 146 retrospective, 165 prospective, 311 total

^p^ Missing data: 78 retrospective, 95 prospective, 173 total

^q^ Missing data: 9 retrospective, 31 prospective, 40 total

^r^ Missing data: 15 retrospective, 16 prospective, 31 total

^s^ Safety analysis: 4 retrospective and 2 prospective patients were included although they met ≥ 1 exclusion criteria and/or not all inclusion criteria

^t^ Including restart of ibrutinib therapy after permanent discontinuation of ibrutinib (i.e. for more than three months)

### Effectiveness

**Fig. 1:**
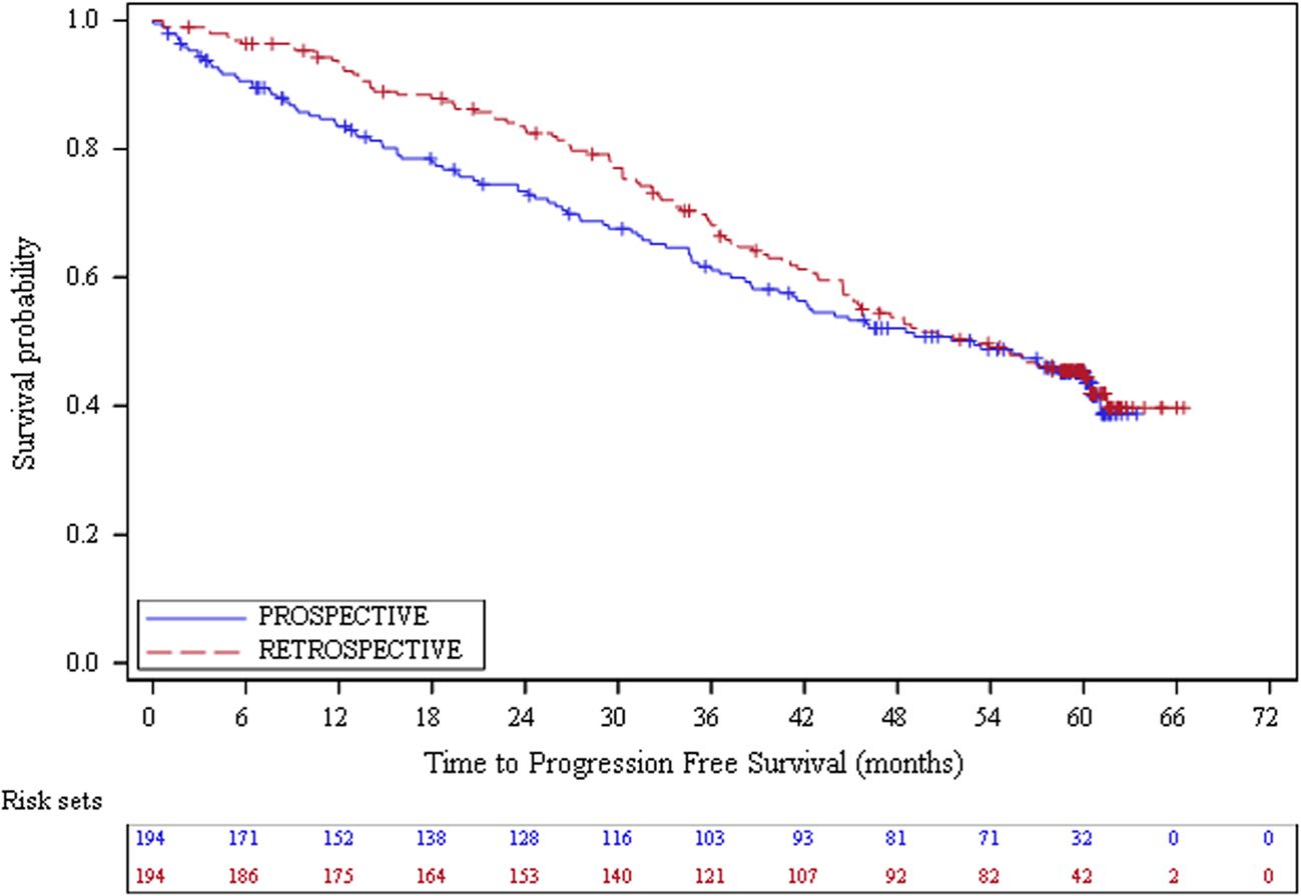
Progression-free survival for CLL patients by type of inclusion (Effectiveness population, *N* = 388)

**Fig. 2:**
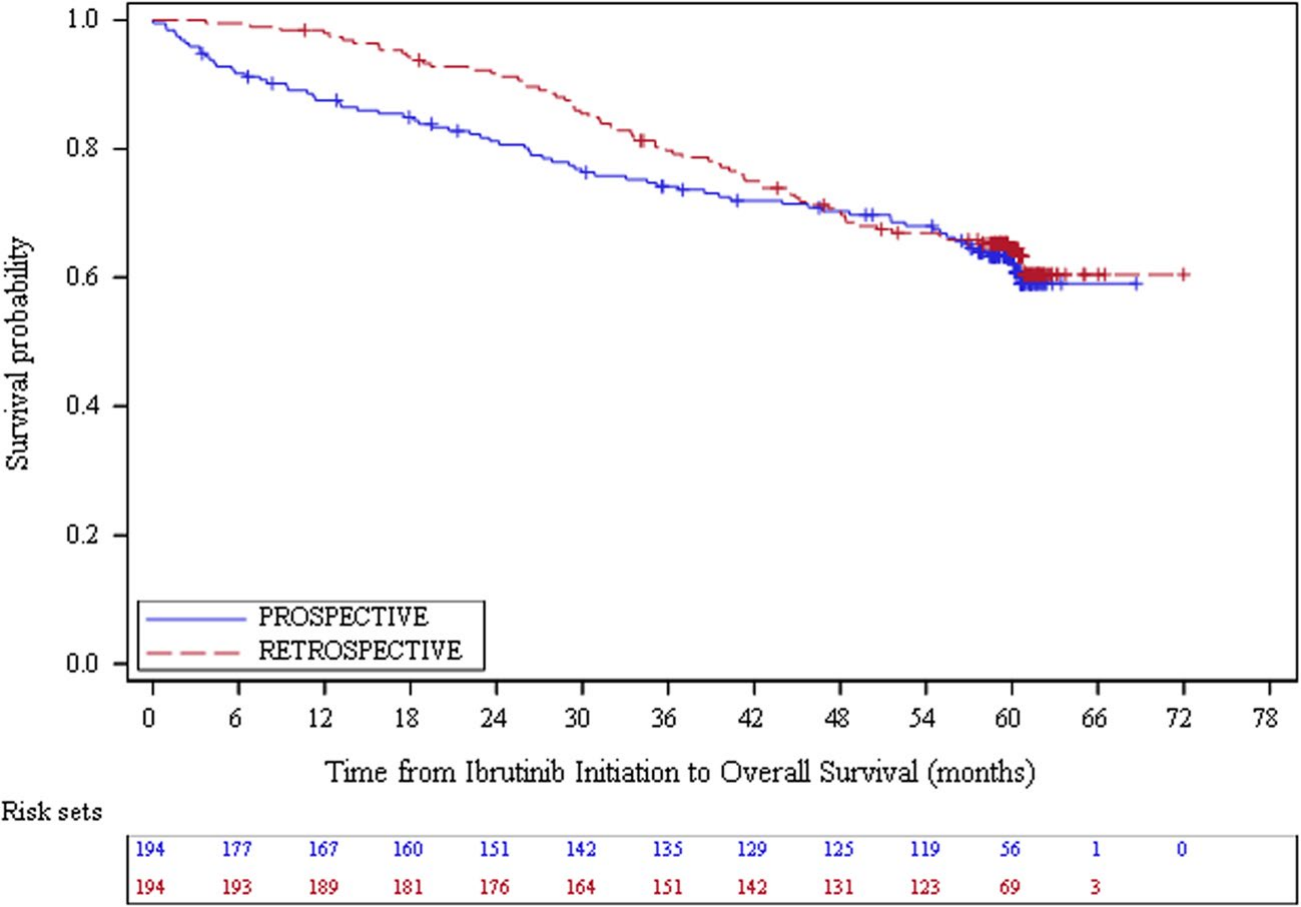
Time from ibrutinib initiation to overall survival for CLL patients by type of inclusion (Effectiveness population, *N* = 388)

**Fig. 3:**
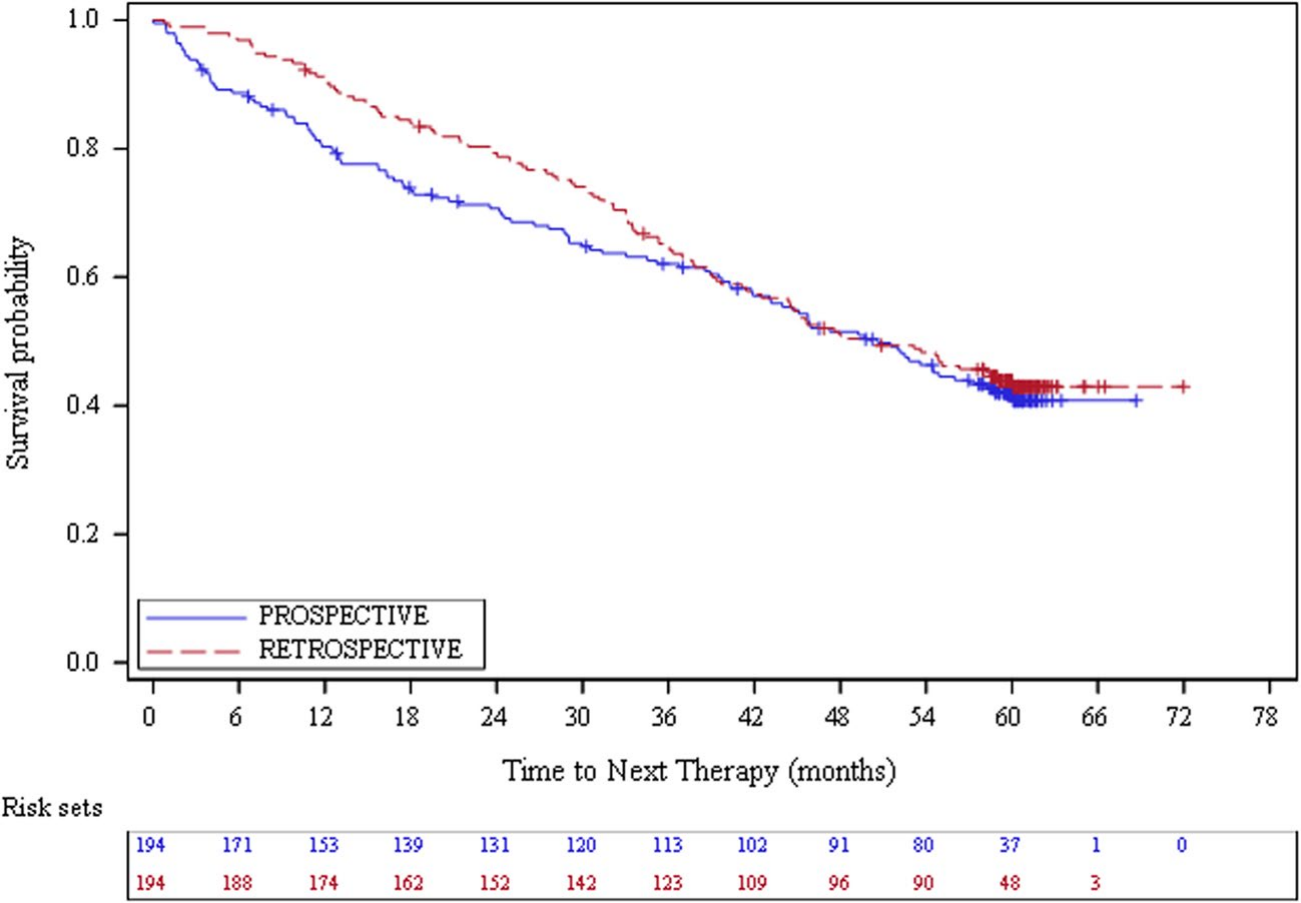
Time to next therapy, excluding patients restarting ibrutinib as subsequent therapy, for CLL patients by type of inclusion (Effectiveness population, *N* = 388)

For retrospective patients, the median follow-up duration was 59.2 (range: 3.7 – 72.0) months with a median PFS of 53.1 (95% CI: 44.5 – 60.5) months (Table 2). PFS rates were 93.2%, 68.1% and 45.5% at month 12, 36 and 60 respectively (Fig. 1). The median OS was not reached (Table 2 and Fig. 2). The OS rates were 97.9%, 79.7% and 64.5% at month 12, 36 and 60 respectively. The median DOR was 59.5 (95% CI: 56.6 – NA) months (Table 2 and Online Resource 3). The median time to first response, best response and next therapy were 2.8 (95% CI: 2.4–3.0), 8.4 (95% CI: 6.7 – 9.4) and 50.1 (95% CI: 41.9 – 60.1) months (Table 2, Online Resources 4 and 5, and Fig. 3). By 60 months, 96.8% of the retrospective patients had a response to ibrutinib treatment: 40.7% had a complete response and 56.1% a partial response (Table 2). The disease progressed in 34.0% of the cases (until month 60).

**Table 2:** Survival, best response and treatment modifications by type of inclusion

Effectiveness population		RETRO (*N* = 194)	PRO (*N* = 194)
**Survival**			
Median follow-up duration (range), months^a^		59.24 (3.7–72.0)	58.53 (0.1–68.7)
Median PFS (95% CI), months		53.06 (44.52–60.45)	52.93 (40.34–60.58)
Median OS (95% CI) ^b^, months		Not reached	Not reached
Median DOR (95% CI), months		59.50 (56.61-NA)	Not reached
Median TTBR (95% CI), months		8.44 (6.74–9.43)	8.21 (5.03–10.55)
Median TTFR (95% CI), months		2.76 (2.43–2.99)	2.76 (2.60–2.92)
Median TTNT (95% CI), months		50.14 (41.86–60.06)	50.63 (41.89–58.28)
**Best response at 60 months, N (%)**^**c**^		189	178
	Overall response	183 (96.8)	172 (96.6)
	CR	77 (40.7)	68 (38.2)
	PR^d^	106 (56.1)	104 (58.4)
	Stable disease	4 (2.1)	2 (1.1)
	Disease progression	2 (1.1)	4 (2.2)
**Best response at 60 months for previously untreated patients with del17p and/or TP53 mutation, N (%)**		24	33
	Overall response	24 (100.0)	33 (100.0)
	CR	8 (33.3)	15 (45.5)
	PR^d^	16 (66.7)	18 (54.5)
	Stable disease	–	–
	Disease progression	–	–
**Best response at 60 months for previously treated patients with del17p and/or TP53 mutation, N (%)**^**e**^		58	38
	Overall response	58 (100.0)	34 (89.5)
	CR	22 (37.9)	15 (39.5)
	PR^d^	36 (62.1)	19 (50.0)
	Stable disease	–	2 (5.3)
	Disease progression	–	2 (5.3)
**Dose reductions**			
Patients with no ibrutinib dose reduction		*N* = 124	*N* = 122
	Median PFS (95% CI), months	49.35 (44.45–61.54)	52.93 (30.85-NA)
	Treatment discontinuation, N (%)	78 (62.9)	84 (68.9)
Patients with at least one ibrutinib dose reduction^f^		*N* = 70	*N* = 72
	Median PFS (95% CI), months	55.23 (39.66-NA)	49.08 (40.34-NA)
	Treatment discontinuation, N (%)	46 (65.7)	52 (72.2)
**Safety population**		**RETRO (*N*** **= 198)**	**PRO (*N*** **= 196)**
**Dose reductions**			
Time to dose reduction as first dose modification^g^		*N* = 43	*N* = 51
	Median time (range), months	7.39 (0.39–60.88)	9.30 (0.39–57.43)
**Permanent discontinuation**			
Time to permanent discontinuation^h^		*N* = 119	*N* = 127
	Median time (range), months	28.65 (0.7–62.8)	18.00 (0.1–61.1)

*Abbreviations*: *CI* Confidence Interval, *CLL* Chronic Lymphocytic Leukaemia, *CR* Complete Response, *DOR* Duration of Response, *NA* Not Available, *OS* Overall Survival, *PFS* Progression-Free Survival, *PR* Partial Response, *PRO* Prospective, *RETRO* Retrospective, *SD* Standard Deviation, *TTBR* Time to Best Response, *TTFR* Time to First Response, *TTNT* Time to Next Therapy

^a^ Calculated as the duration from ibrutinib initiation until the end of study date

^b^ From ibrutinib initiation to OS

^c^ 5 missing for retrospective patients, 15 missing and 1 not evaluable for prospective patients

^d^ Including partial response with lymphocytosis

^e^ 1 missing for retrospective patients and 2 missing for prospective patients

^f^ PFS of patients with at least one ibrutinib dose reduction versus PFS of patients with no ibrutinib dose reduction: *p* = 0.7971 for retrospective. patients and *p* = 0.3163 for prospective patients

^g^ Calculated as the duration from ibrutinib initiation to dose reduction as first modification

^h^ Calculated as the duration from ibrutinib initiation to permanent discontinuation

For prospective patients, the median follow-up duration was 58.5 (range: 0.1–68.7) months with a median PFS of 52.9 (95% CI: 40.3–60.6) months (Table 2). PFS rates were 83.5%, 61.1% and 45.1% at month 12, 36 and 60 respectively (Fig. 1). The median OS and the median DOR were not reached (Table 2, Fig. 2 and Online Resource 3). The OS rates were 87.6%, 74.2% and 63.3% at month 12, 36 and 60 respectively (Fig. 2). The median time to first response, best response and next therapy were 2.8 (95% CI: 2.6–2.9), 8.2 (95% CI: 5.0–10.6) and 50.6 (95% CI: 41.9–58.3) months respectively (Table 2, Online Resources 4 and 5, and Fig. 3). By 60 months, 96.6% of the prospective patients had a response to ibrutinib treatment: 38.2% had a complete response and 58.4% a partial response (Table 2). The disease progressed in 29.4% of the cases (until month 60).

**Fig. 4:**
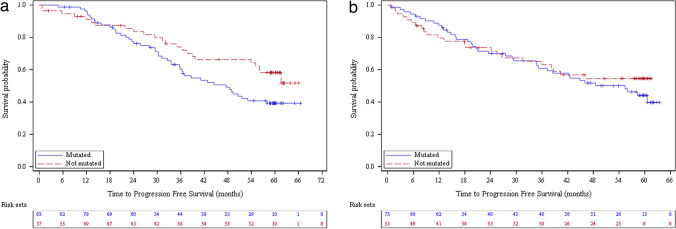
Progression-free survival for CLL patients according to mutation status (del17p and/or TP53) for retrospective patients (**a**) and prospective patients (**b**) (Effectiveness population, *N* = 388)

When mutation status (del17p and/or TP53) was taken into account, the median PFS for retrospective patients with a mutation was 47.5 (95% CI: 35.8 – NA) months but was not reached for those without mutation (Fig. 4A). PFS rates were 96.3%, 60.4% and 39.3% at month 12, 36 and 60 respectively for those with mutation versus 91.1%, 74.0% and 58.2% for those without. For prospective patients, the median PFS for those with a mutation was 55.4 (95% CI: 34.8 – NA) months but was not reached for those without mutation (Fig. 4B). PFS rates were 87.4%, 60.9% and 44.1% at month 12, 36 and 60 respectively for those with mutation versus 79.6%, 63.1% and 54.5% for those without.

### Dose reduction of ibrutinib

For 91.4% of the retrospective patients and 91.3% of the prospective patients, the daily dose of ibrutinib at initiation was 420 mg with a median overall treatment duration of 42.1 (range: 0.7–66.5) months for retrospective and 39.2 (range: 0.0–63.5) months for prospective patients. For retrospective patients, the median duration of treatment by ibrutinib until inclusion was 9.0 (range: 1.0–24.6) months. More than half of the patients had no dose modifications (56.1% of the retrospective and 58.7% of the prospective patients).

**Fig. 5:**
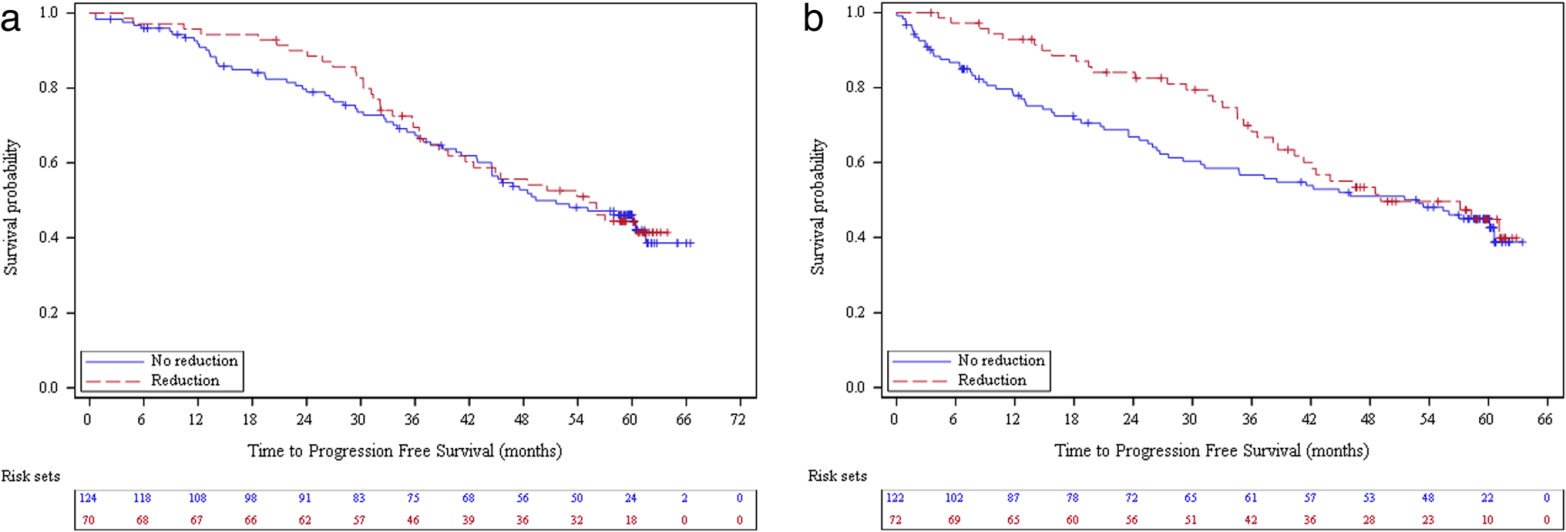
Progression-free survival for CLL patients with at least one ibrutinib dose reduction versus no dose reduction for retrospective patients (**a**) and prospective patients (**b**) (Effectiveness population, *N* = 388)

For those who had at least one dose modification (43.9% retrospective and 41.3% prospective), toxicity was the main reason of dosing change (56.3% retrospective and 64.2% prospective). Among those who had at least one dose reduction (36.1% retrospective and 37.1% prospective patients), the mean number of dose reduction was 1.5 (SD = 0.7) for retrospective patients and 1.3 (SD = 0.7) for prospective patients with a mean duration of 10.3 months (SD = 10.3) and 8.8 months (SD = 9.0) respectively. The PFS for patients with at least one dose reduction was 55.2 (95% CI: 39.7 – NA) months for the retrospective group and 49.1 (95% CI: 40.3 – NA) months for the prospective group versus 49.4 (95% CI: 44.5–61.5) and 52.9 (95% CI: 30.9-NA) months, respectively, for those with no dose reduction (63.9% retrospective and 62.9% prospective patients) (*p* = 0.7971 retrospective and *p* = 0.3163 prospective) (Table 2, Fig. 5A and B). The median time between treatment instauration and first dose reduction as first dose modification was 7.4 (range: 0.4–60.9) months for retrospective patients (*N* = 43) and 9.3 (range: 0.4–57.4) months for prospective patients (*N* = 51) (Table 2).

Overall, permanent ibrutinib discontinuation was observed in 119 retrospective patients (60.1%) and in 127 prospective patients (64.8%) (Table 2). The median time from ibrutinib initiation to permanent discontinuation was 28.7 (range: 0.7–62.8) months for retrospective patients and 18.0 (range: 0.1–61.1) months for prospective patients, and the main reasons for discontinuation were toxicity (43.5% retrospective and 42.0% prospective), disease progression (33.0% retrospective and 32.8% prospective) and death (5.2% retrospective and 10.1% prospective). Among retrospective patients who discontinued ibrutinib because of toxicity (*N* = 50), 5 (10.0%) patients had no prior line of treatment, 18 (36.0%) one prior line and 27 (54.0%) at least two prior lines. Among prospective patients (*N* = 50), 7 (14.0%) had no prior line of treatment, 21 (42.0%) one prior line and 22 (44.0%) two prior lines or more. After ibrutinib treatment, less than half (41.9% of the retrospective and 33.2% of the prospective patients) received a subsequent therapy (Table 1). The most frequent subsequent treatment was Venetoclax ± Rituximab for 54.2% of the retrospective and for 49.2% of the prospective patients. Ibrutinib was retaken by 14.5% of the retrospective and by 18.4% of the prospective patients.

### Safety

Almost all patients (85.9% of the retrospective and 99.5% of the prospective patients) had at least one TEAE (Table 3). For 17.7% of the retrospective and for 30.1% of the prospective patients, TEAEs were related to ibrutinib and considered by the investigators as serious. The most frequent TEAEs of interest were infections (57.6%), neoplasms (18.7%), diarrhoea (16.2%), and hypertension (14.6%) for retrospective patients and infections (71.4%), diarrhoea (28.6%), arthralgia/myalgia (26.5%) and neoplasms (26.0%) for prospective patients. A total of 16 retrospective patients (8.1%) and 22 prospective patients (11.2%) had atrial fibrillation or flutter. Regarding treatment-emergent bleeding event, 28.8% of the retrospective patients and 54.1% of the prospective patients had such events, and more bleeding events were noticed among patients under antithrombotic treatment. Bleeding events were considered as major for 2.0% of the retrospective patients and for 8.2% of the prospective patients. A total of 14.6% of the retrospective and 22.4% of the prospective patients had at least one AE leading to death with the most important preferred terms being general physical health deterioration for both groups (3.5% for retrospective and 2.0% for prospective patients) as well as septic shock (2.0%), sepsis (2.0%) and Richter’s transformation (2.0%) for prospective patients (Table 4). Other AEs leading to death are detailed in Table 4.

**Table 3:** Adverse events (AEs) and treatment-emergent adverse events (TEAEs) of interest by type of inclusion (Safety population, *N* = 394)

		RETRO after inclusion (*N* = 198)^a^	PRO (*N* = 196)^a^
**Patients with at least one AE, N (%)**		175 (88.4)	195 (99.5)
**Patients with TEAE (any severity), N (%)**			
	≥ 1 TEAE	170 (85.9)	195 (99.5)
	≥ 1 serious TEAE	100 (50.5)	142 (72.4)
	≥ 1 severe TEAE	98 (49.5)	143 (73.0)
	≥ 1 TEAE related to ibrutinib^b^	135 (68.2)	181 (92.3)
	≥ 1 serious TEAE related to ibrutinib^c^	35 (17.7)	59 (30.1)
**Patients with treatment-emergent bleeding events, N (%)**			
	≥ 1 bleeding	57 (28.8)	106 (54.1)
	≥ 1 major bleeding	4 (2.0)	16 (8.2)
	≥ 1 bleeding while on antithrombotic treatment^d^	16/39 (41.0)	34/45 (75.6)
	≥ 1 major bleeding while on antithrombotic treatment^d^	2/39 (5.1)	7/45 (15.6)
	≥ 1 bleeding while NOT on antithrombotic treatment^e^	41/159 (25.8)	72/151 (47.7)
	≥ 1 major bleeding while NOT on antithrombotic treatment^e^	2/159 (1.3)	9/151 (6.0)
**Patients with TEAE of interest (any severity), N (%)**			
	≥ 1 infection	114 (57.6)	140 (71.4)
	≥ 1 neoplasm	37 (18.7)	51 (26.0)
	≥ 1 diarrhoea	32 (16.2)	56 (28.6)
	≥ 1 arthralgia/myalgia	27 (13.6)	52 (26.5)
	≥ 1 atrial fibrillation or flutter	16 (8.1)	22 (11.2)
	≥ 1 hypertension	29 (14.6)	29 (14.8)
	≥ 1 rash	16 (8.1)	22 (11.2)
	≥ 1 Richter’s transformation	2 (1.0)	5 (2.6)

*Abbreviations*: *AE* Adverse Event, *CLL* Chronic Lymphocytic Leukaemia, *PRO* Prospective, *RETRO* Retrospective, *TEAE* Treatment-Emergent Adverse Event

^a^ 4 retrospective and 2 prospective patients were included for the safety analysis although they met ≥ 1 exclusion criteria and/or not all inclusion criteria

^b^ 166 (83.8%) retrospective patients had at least one TEAE related to ibrutinib before inclusion

^c^ 16 (8.1%) retrospective patients had at least one serious TEAE related to ibrutinib before inclusion

^d^ Percentage are calculated over the number of patients with antithrombotic treatment (*N* = 39 for the retrospective patients; *N* = 45 for the prospective patients)

^e^ Percentage are calculated over the number of patients without antithrombotic treatment (*N* = 159 for the retrospective patients; *N* = 151 for the prospective patients)

**Table 4:** Adverse events (AEs) leading to death by type of inclusion (Safety population, *N* = 394)

		RETRO after inclusion (*N* = 198)^a,b^	PRO (*N* = 196)^a^
**Patients with AEs leading to death, N (%)**			
Patients with at least one AE leading to death		29 (14.6)	44 (22.4)
	Still under treatment at the time of death	9 (31.0)	18 (40.9)
Patients with at least one TEAE leading to death		26 (13.1)	41 (20.9)
**Patients with at least one AE leading to death classified by SOC and PT, N (%)**			
**SOC**^**c**^	**PT**^**c**^		
≥ 1 cardiac disorder		2 (1.0)	1 (0.5)
	Cardiac failure	–	1 (0.5)
	Cardiorespiratory arrest	1 (0.5)	–
	Congestive cardiomyopathy	1 (0.5)	–
≥ 1 general disorder and administration site condition		9 (4.5)	7 (3.6)
	General physical health deterioration	7 (3.5)	4 (2.0)
	Death	2 (1.0)	3 (1.5)
≥ 1 hepatobiliary disorder		–	1 (0.5)
	Drug-induced liver injury	–	1 (0.5)
≥ 1 infection and infestation		8 (4.0)	15 (7.7)
	Septic choc	2 (1.0)	4 (2.0)
	Covid-19	1 (0.5)	3 (1.5)
	Sepsis	–	4 (2.0)
	Cerebral aspergillosis	2 (1.0)	–
	Meningitis	–	2 (1.0)
	Atypical mycobacterial pneumonia	–	1 (0.5)
	Bronchitis	1 (0.5)	–
	Fungaemia	–	1 (0.5)
	Fungal infection	–	1 (0.5)
	Pneumocystis jirovecii pneumonia	–	1 (0.5)
	Pneumonia	1 (0.5)	–
	Pulmonary mucormycosis	–	1 (0.5)
	Rhinocerebral mucormycosis	–	1 (0.5)
	Urosepsis	1 (0.5)	–
≥ 1 injury, poisoning and procedural complication		–	1 (0.5)
	Subdural haematoma	–	1 (0.5)
≥ 1 metabolism and nutrition disorder		–	1 (0.5)
	Tumour Lysis Syndrome	–	1 (0.5)
≥ 1 neoplasm benign, malignant and unspecified (incl. cysts and polyps)		7 (3.5)	11 (5.6)
	Richter’s transformation	1 (0.5)	4 (2.0)
	Prostate cancer	1 (0.5)	1 (0.5)
	B-cell lymphoma	–	1 (0.5)
	Breast cancer metastatic	–	1 (0.5)
	Chronic lymphocytic leukaemia	–	1 (0.5)
	Colorectal cancer metastatic	–	1 (0.5)
	Cutaneous t-cell lymphoma	1 (0.5)	–
	Lung neoplasm malignant	1 (0.5)	–
	Metastases to central nervous system	1 (0.5)	–
	Metastatic bronchial carcinoma	1 (0.5)	–
	Neuroendocrine carcinoma	1 (0.5)	–
	Oespophageal squamous cell carcinoma stage 0	–	1 (0.5)
	Pancreatic carcinoma	1 (0.5)	–
	Transitional cell carcinoma	–	1 (0.5)
≥ 1 nervous system disorder		2 (1.0)	2 (1.0)
	Central nervous system lesion	1 (0.5)	-
	Cerebellar haematoma	–	1 (0.5)
	Cerebral haemorrhage	–	1 (0.5)
	Intraventicular haemorrage	1 (0.5)	–
≥ 1 respiratory, thoracic and mediastinal disorder		3 (1.5)	4 (2.0)
	Lung disorder	–	3 (1.5)
	Acute respiratory distress syndrome	1 (0.5)	–
	Pneumonitis	1 (0.5)	–
	Pulmonary embolism	1 (0.5)	–
	Respiratory distress	–	1 (0.5)
≥ 1 vascular disorder		–	1 (0.5)
	Infarction	–	1 (0.5)
**AEs leading to death: relationship with ibrutinib, N (%)**		***N*** **=** **32**	***N*** **=** **47**^**d**^
	Doubtful	3 (9.4)	3 (6.4)
	Possible related	1 (3.1)	5 (10.6)
	Probably related	2 (6.3)	3 (6.4)
	Very likely related	2 (6.3)	2 (4.3)
	Not related	24 (75.0)	34 (72.3)

*Abbreviations*: *AE* Adverse Event, *CLL* Chronic Lymphocytic Leukaemia, *PRO* prospective, *PT* Preferred term, *RETRO* retrospective, *SOC* System Organ Class, *TEAE* Treatment-Emergent Adverse Event

^a^ 4 retrospective and 2 prospective patients were included for the safety analysis although they met ≥ one exclusion criteria and/or not all inclusion criteria

^b^ 2 retrospective patients died before inclusion. For one patient, the AEs leading to death were cytopenia, dyspnoea and general physical health deterioration. For the second patient, the AE leading to death was general physical health deterioration

^c^ 32 AEs leading to death in 29 retrospective patients and 48 AEs leading to death in 44 prospective patients. Patients could be in more than one SOC and PT if multiple causes of death

^d^ 1 missing for prospective patients

## Discussion

Although clinical trials have always been the gold standard of proof regarding effectiveness and safety of new drugs, there is nowadays a great interest in real-world research since they represent patients in real-life settings. To our knowledge, FIRE was the largest French real-word study that assessed the effectiveness and safety of ibrutinib, in accordance with the French marketing authorization in 2016, for the treatment of CLL/SLL in patients who received at least one prior line of treatment, or who were previously untreated and had a del17p and/or TP53 mutation unsuitable for chemoimmunotherapy. In this extensive study, set up in 65 centres, 388 CLL/SLL patients (194 retrospective and 194 prospective) were included in the effectiveness population and followed-up for five years.

Our results are consistent with previous effectiveness findings [17, 18]. In a real-world multicenter retrospective study, conducted on 205 CLL patients treated with ibrutinib, the 12-months PFS and OS rates were 86.3% and 88.8% respectively [17]. In another study on long-term efficacy and safety with a median follow-up of 5 years, in which 31 treatment-naïve and 101 R/R patients were included, the median PFS in R/R patients was 51 months with a 5-year PFS rate of 44% [18]. The median OS was not reached and the OS rate at 5 years was 60%. In a UK/Ireland-based study, the one-year OS was 83.8% [19]. In the clinical trial RESONATE, only R/R CLL patients were included. When comparing the results at similar timepoints between RESONATE and FIRE, the one-year PFS and OS rates in RESONATE (84% and 90% respectively) as well as the 5-year PFS (40.0%) were similar to those of FIRE [7, 8, 20]. The ORR was also similar: 91% in RESONATE vs. 96.8% and 96.6% for retrospective and prospective patients, respectively, in FIRE [8] (Online Resource 6). However when comparing median PFS and OS, those in RESONATE were lower: 44.1 (95% CI: 38.5–56.2) months for the median PFS and 67.7 (95% CI: 61.0 – not reached) months for the median OS [8] (Online Resource 6). One explanation could be the longer follow-up period in RESONATE (6 years vs. 5 years). However, taking the fact that our results are included in the confidence intervals of the PFS and OS of RESONATE, our finding are consistent. Therefore, although the FIRE population is slightly different than the population in RESONATE (e.g. age, ECOG PS, mutations status, number of prior therapies), it is reassuring to see that our effectiveness results are similar to the results of clinical trials.

Our efficacy results showed that patients with at least one dose reduction had a similar PFS than patients with no dose reduction, supporting the fact that CLL patients in France are well managed, follow-up and treated. Our results not only confirm those of previous real-world studies [19, 21, 22] but also encourage the idea that ibrutinib can still be administrated to patients presenting AEs. Therefore, if physicians need to modify the dose because of an AE, dose reduction may be the best option. Suggesting dose reductions to patients in need of dose modifications will thus reduce treatment discontinuation, increase patient adherence, improve patients outcome and on a long-term strategy decrease financial and economic burden. However, to obtain the best benefit from ibrutinib, it is important to promptly identify and manage AEs, and understand specific AEs that can be associated with the need for dose reductions.

The median time to first dose reduction was assessed in a retrospective chart review on first line and R/R CLL patients treated with ibrutinib either in academic practice or community network [23]. Their results (median time of 3.6 months overall) were lower than ours (7.4 and 9.3 months for retrospective and prospective patients respectively). Furthermore, a review on ibrutinib dose modifications in the management of CLL mentioned that in real-world settings, dose reductions over the first year was often noticed [24]. However, addressing the question of time in dose reduction still remains rare and unclear. Therefore, further research on this topic is necessary in order to better understand the role of time in dose reductions and ibrutinib outcome.

Moreover, our results showed that among patients who discontinued ibrutinib, toxicity was the main reason for around 40.0% of them. These results were similar to the one found in a Swedish retrospective study: 40.4% (19/47) [21]. However, a Danish retrospective study showed a higher rate: 54.7% (47/86) [17]. In RESONATE, the discontinuation rate due to toxicity was much lower 21.1% (32/152) (Online Resource 6). One explanation to this lower discontinuation rate due to AEs compared to the FIRE study could be that RESONATE is a clinical trial with eligibility criteria which promote inclusion of selected patient. Closer monitoring in clinical trials could be also another explanation. Although our results on discontinuation rates due to AEs differed from the one found in RESONATE, they illustrate the need of real-world research on long-term safety on heterogeneous population.

Among AEs noticed in our study, patients reported low rate of major bleeding events (2.0% retrospective and 8.2% prospective). This rate was five times less for retrospective patients but similar for prospective patients than the rate reported in RESONATE (10.0%) [8]. Of note, in FIRE, more patients had a bleeding / major bleeding event when they were under antithrombotic treatment. Explanations could be that bleeding events are side effects of such treatments, and in RESONATE, patients under anticoagulation containing warfarin were excluded. In addition, the rate of atrial fibrillation was similar for patients in the two studies (FIRE: 8.1% for retrospective and 11.2% for prospective patients; RESONATE: 12.0%) but the rate of hypertension was lower in FIRE than in RESONATE (FIRE: 14.6% for retrospective and 14.8% for prospective; RESONATE: 21.0%) [8]. Nevertheless, it is reassuring to see that there was no new AE observed and that the safety profile of ibrutinib in our study seems to be consistent with previous studies.

Finally, while the development and distribution of ibrutinib has transformed treatment expectations for CLL patients, at the time when our study was set up, in 2016, ibrutinib was used only in monotherapy, and therefore, patients usually needed to be continuously treated until disease progression or onset of AEs. Being continuously treated has several consequences, and hence, to reverse the situation and stop treatment once the illness is in remission, fixed-duration ibrutinib-combination therapies have been developed and had shown promising results [25, 26]. For instance, the GLOW clinical trial showed a 42-month PFS rate of 74.6% for previously untreated CLL patients under fixed-duration ibrutinib-venetoclax therapy, higher than our results in monotherapy at 42 months (± 60% for retrospective patients and slightly less than 60% for prospective patients), and similar findings on fatal AEs [25]. However, although GLOW is a clinical trial and had different inclusion criteria than ours, and therefore direct comparison should be done with caution, positive impacts of ibrutinib-combination treatment at fixed-durations still seem to add a real value to ibrutinib treatment as monotherapy which are encouraging for both patients and further research.

Our study has several strengths and limitations. First, FIRE was the largest real-word study on the effectiveness and safety of ibrutinib in France. Second, because of its real-world design, effectiveness and safety parameters were presented through descriptive data in a “real-life condition”, and therefore, our results complement those of clinical trials. Moreover, all consecutive patients who met the eligibility criteria and who had therapy-demanding disease were considered for inclusion in order to reduce selection bias. However, there might have been a bias in effectiveness results between retrospective and prospective patients since retrospective patients who died before enrolment were not included. Therefore, retrospective patients who were included in the study should be considered in “better” health than prospective patients. Nevertheless, it is reassuring to see that the results between the two groups are quite similar. In addition, because of the exclusion of retrospective patients who died before enrolment, it was difficult to pull data of retrospective patients together with the data of prospective patients. Furthermore, the number of AEs for retrospective patients have been underestimated since TEAEs that occurred before inclusion and that were not related to ibrutinib were not collected for these patients. Finally, our focus was on the effectiveness and safety profile of ibrutinib. Therefore, other aspects such as the quality of life of patients under ibrutinib were not considered in this article. Although data on quality of life might have been informative and complement the findings of this article, this whole topic will be discussed in a separate paper.

## Conclusion

In conclusion, FIRE was a large real-word study, with a long follow-up period, that included many centres and CLL patients, and showed the effectiveness of ibrutinib on the PFS and OS, as well as on other effectiveness parameters. Dose modifications were mainly attributed to toxicity. However, it is reassuring to see that patients who had at least a dose reduction had a similar PFS than patients with no dose reduction, implying the fact that ibrutinib can still be administrated in case of AEs. No additional safety concerns than those already mentioned in other studies could be noticed. Finally, our results not only complement those of clinical trials, but they are also consistent with both results of clinical trials and other real-world studies.

## Supplementary Information

Below is the link to the electronic supplementary material.

Supplementary file1 (DOCX 209 KB)

## Data Availability

The data-sharing policy of the Janssen Pharmaceutical Companies of Johnson & Johnson is available at www.janssen.com/clinical-trials/transparency. Requests for access to the data from selected studies can be submitted through the Yale Open Data Access (YODA) Project site at yoda.yale.edu.
