# Supplementary material for: Final results on effectiveness and safety of Ibrutinib in patients with chronic lymphocytic leukemia from the non-interventional FIRE study
Source: Ann Hematol. 2024 Mar 6;104(2):1079–93. doi: 10.1007/s00277-024-05666-3 (PMC11971162; doi:10.1007/s00277-024-05666-3)
Supplement: Supplementary file 1 — Supplementary file1 (DOCX 209 KB) [file 277_2024_5666_MOESM1_ESM.docx]

**ONLINE RESOURCE**

**Final results on effectiveness and safety of Ibrutinib in patients with chronic lymphocytic leukemia from the non-interventional FIRE study.**

**Caroline DARTIGEAS^1a^, Anne QUINQUENEL^2^, Loïc YSEBAERT^3^, Marie-Sarah DILHUYDY^4^, Bruno ANGLARET^5^, Borhane SLAMA^6^, Katell LE DU^7^, Stéphanie TARDY^8^, Emmanuelle TCHERNONOG^9^, Hubert ORFEUVRE^10^, Laurent VOILLAT^11^, Stéphanie GUIDEZ^12^ Jean-Valère MALFUSON^13^, Sandrine DUPUIS^14^, Marine DESLANDES^14^, Pierre FEUGIER^15^, Véronique LEBLOND^16^, on behalf of** **the FIRE Investigators Group**

^1^ CHRU Hôpitaux de Tours, Tours, France

^2^ CHU de Reims, Reims, France

^3^ IUCT Oncopôle, CHU de Toulouse, Toulouse, France

^4^ Hôpital Haut-Lévêque, Bordeaux, France

^5^ CH de Valence, Valence, France

^6^ CH Henri Duffaut, Avignon, France

^7^ Hôpital Privé du Confluent, Nantes, France

^8^ CH Annecy Genevois, Annecy, France

^9^ CHU de Montpellier, Montpellier, France

^10^ CH de Bourg-en-Bresse, Bourg-en-Bresse, France

^11^ CH William Morey, Chalon-sur-Saone, France

^12^ CHU de Poitiers, Poitiers, France

^13^ HIA Percy, Clamart, France

^14^ Janssen France, Issy-les-Moulineaux, France

^15^ Hôpitaux de Brabois, CHU de Nancy, Nancy, France

^16^ AP-HP Hôpital de la Pitié-Salpêtrière, Paris La Sorbonne

**Online Resource 1. Overall design of the study.**


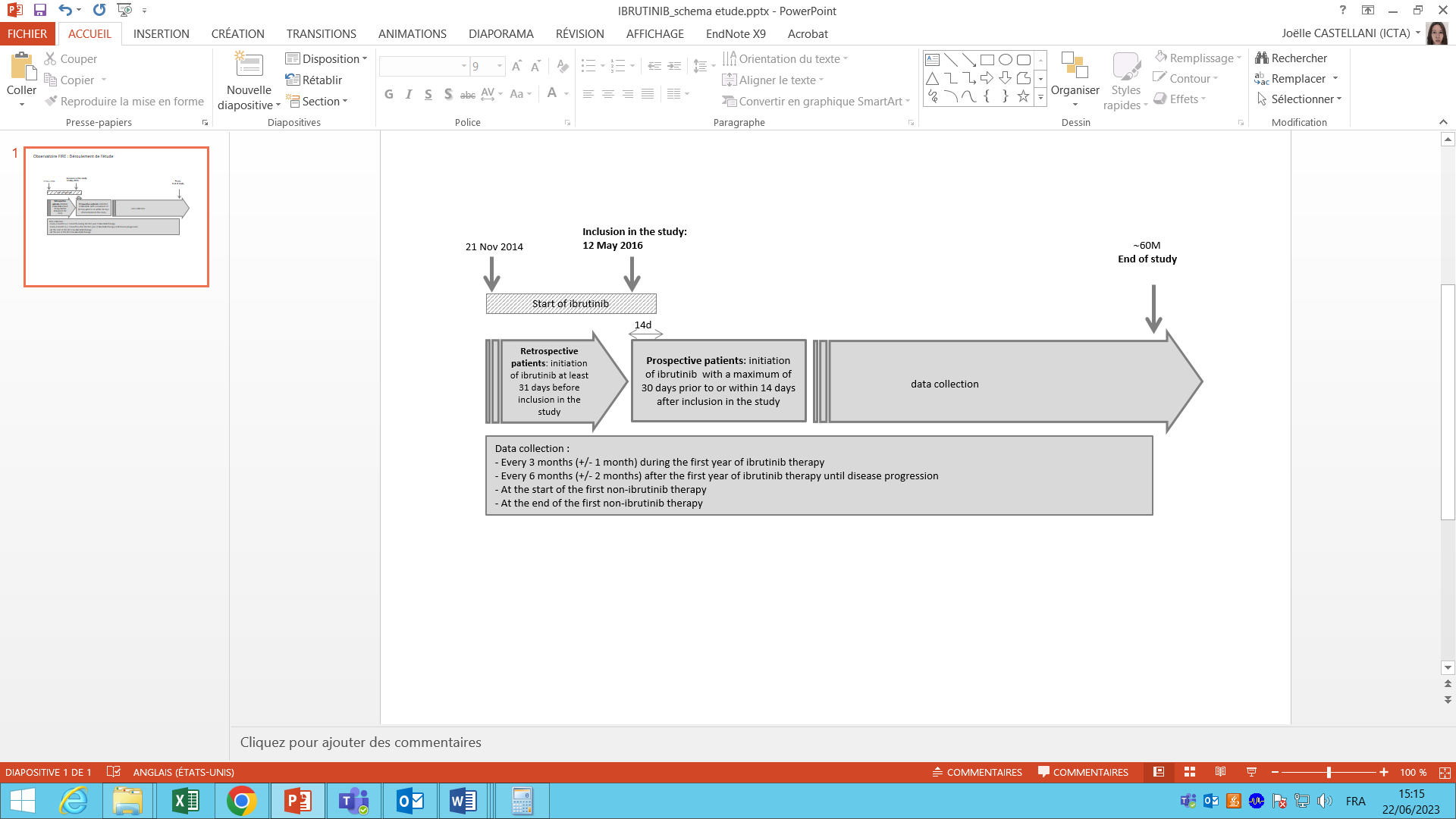


**Online Resource 2. Definitions of the primary and secondary endpoints.**

| **Endpoints** | **Definitions** |
| --- | --- |
| PFS | The time from ibrutinib initiation to progression or death from any cause of death |
|  |  |
| OS | The time from ibrutinib initiation to the date of death from any cause of death |
|  |  |
| DOR | The time from response to ibrutinib to progression or death resulting from progression |
|  |  |
| Time to best response | The time form ibrutinib initiation to the best objective response (complete response, partial response, partial response with lymphocytosis) |
|  |  |
| Time to first response | The time from ibrutinib initiation to the first objective response (complete response, partial response, partial response with lymphocytosis) |
|  |  |
| Time to next treatment | The time from ibrutinib initiation to the start of non-ibrutinib subsequent therapy or death from any cause |
|  |  |

**Online Resource 3. Duration of response for CLL patients by type of inclusion (Effectiveness population, N=388).**

**Online Resource 4. Time to first response for CLL patients by type of inclusion (Effectiveness population, N=388).**

**Online Resource 5. Time to best response for CLL patients by type of inclusion (Effectiveness population, N=388).**

**Online Resource 6. Comparison of the results between RESONATE and FIRE Studies**

|  |  | **RESONATE^a^**  **(N=195)** | **FIRE** | |
| --- | --- | --- | --- | --- |
|  |  |  | **Retro (N=194)** | **Pro (N=194)** |
| **Study population** |  |  |  | |
| Patients CLL/SLL |  | R/R CLL (L2+) | L1 (del17p) and L2+ | |
| Follow-up, months | Median (range) | 65.3 (0.3−71.6) | 59.24 (3.7-72.0) | 58.53 (0.1-68.7) |
| **Patients’ characteristics** |  |  |  |  |
| Sex | Male, N (%) | 129 (66.2) | 122 (62.9) | 136 (70.1) |
| Age, years | Median (range) | 67 (30-86) | 72 (43-91) | 71 (39-93) |
| ECOG PS | 0 | 79 (40.5) | 79 (53.0) | 76 (48.4) |
|  | 1 | 116 (59.5) | 56 (37.6) | 63 (40.1) |
|  | 2 | - | 11 (7.4) | 15 (9.6) |
|  | 3 | - | 3 (2.0) | 3 (1.9) |
| Duration of ibrutinib, months | Median (range) | 41.0 (0.2‐71.1) | 39.2 (0.7-66.5) | 34.5 (0.0-63.5) |
| **Permanent discontinuation** |  |  |  |  |
| Permanent discontinuation | N (%) | 152 (77.9)^b^ | 119 (61.3) | 127 (65.5) |
| Reasons for permanent discontinuation (N, %) | N (%) | 152 | 115 | 119 |
|  | Disease progression | 72 (47.4) | 38 (33.0) | 39 (32.8) |
|  | Adverse event | 32 (21.1) | 50 (43.5) | 50 (42.0) |
|  | Death | 13 (6.7) | 6 (5.2) | 12 (10.1) |
|  | Patient withdrawal / preference | 15 (8.6) | 2 (1.7) | 4 (3.4) |
|  | Investigator decision | 20 (13.2) | 4 (3.5) | 1 (0.8) |
|  | Other | - | 15 (13.0) | 13 (10.9) |
| **Effectiveness outcomes** |  |  |  |  |
| Median PFS (95% CI) |  | 44.1 (38.5-56.2) | 53.06 (44.5-60.5) | 52.93 (40.3-60.6) |
| Median OS (95% CI) |  | 67.7 (61.0-NR) | NR | NR |
| Overall Response Rate |  | 91% | 96.8% | 96.6% |
| *Abbreviations: CI, Confidence Interval; CLL, Chronic Lymphocytic Leukaemia; ECOG PS, Eastern Cooperative Oncology Group Performance Status; NR, Not Reached; PRO, Prospective; Retro, Retrospective; SLL, Small Lymphocytic Lymphoma.*  ^a^ Reference [8].  *^b^ 43 patients who discontinued treatment because of Sponsor’s decision have been excluded: they were still on ibrutinib at the end of the study but had to stop treatment because of study closure.* | | | | |
